# Case Report: Rapid Recognition and Immune Modulation of Secondary HLH Due to Disseminated HSV Infection

**DOI:** 10.3389/fped.2021.681055

**Published:** 2021-07-02

**Authors:** Daniel J. McKeone, Theodore K. M. DeMartini, Robert P. Kavanagh, E. Scott Halstead

**Affiliations:** Penn State University College of Medicine, Hershey, PA, United States

**Keywords:** hemophagocytic lymphohistiocytosis, herpes simplex virus, hyperferritinemic syndrome, multiple organ dysfunction syndrome, immune modulation

## Abstract

We describe the case of a newborn who presented with multiple organ dysfunction syndrome (MODS) and hyperferritinemia, who eventually met criteria for hemophagocytic lymphohistiocytosis (HLH) due to disseminated herpes simplex virus 1 (HSV-1). While the cytokine storm abated after administration of multiple immune modulatory therapies including dexamethasone, etoposide, intravenous immune globulin, anakinra, as well as the interferon gamma antagonist emapalumab, multiple organ dysfunction syndrome progressed. Care was withdrawn after 5 days. Subsequent genetic testing did not reveal any mutations associated with familial HLH. This case highlights that even with appropriate antiviral treatment and immune suppression, disseminated HSV is often fatal. Further study is warranted to determine whether early immune modulatory therapy including interferon gamma blockade can interrupt the HLH inflammatory cascade and prevent progression of MODS.

## Case Report

**Figure 1:**
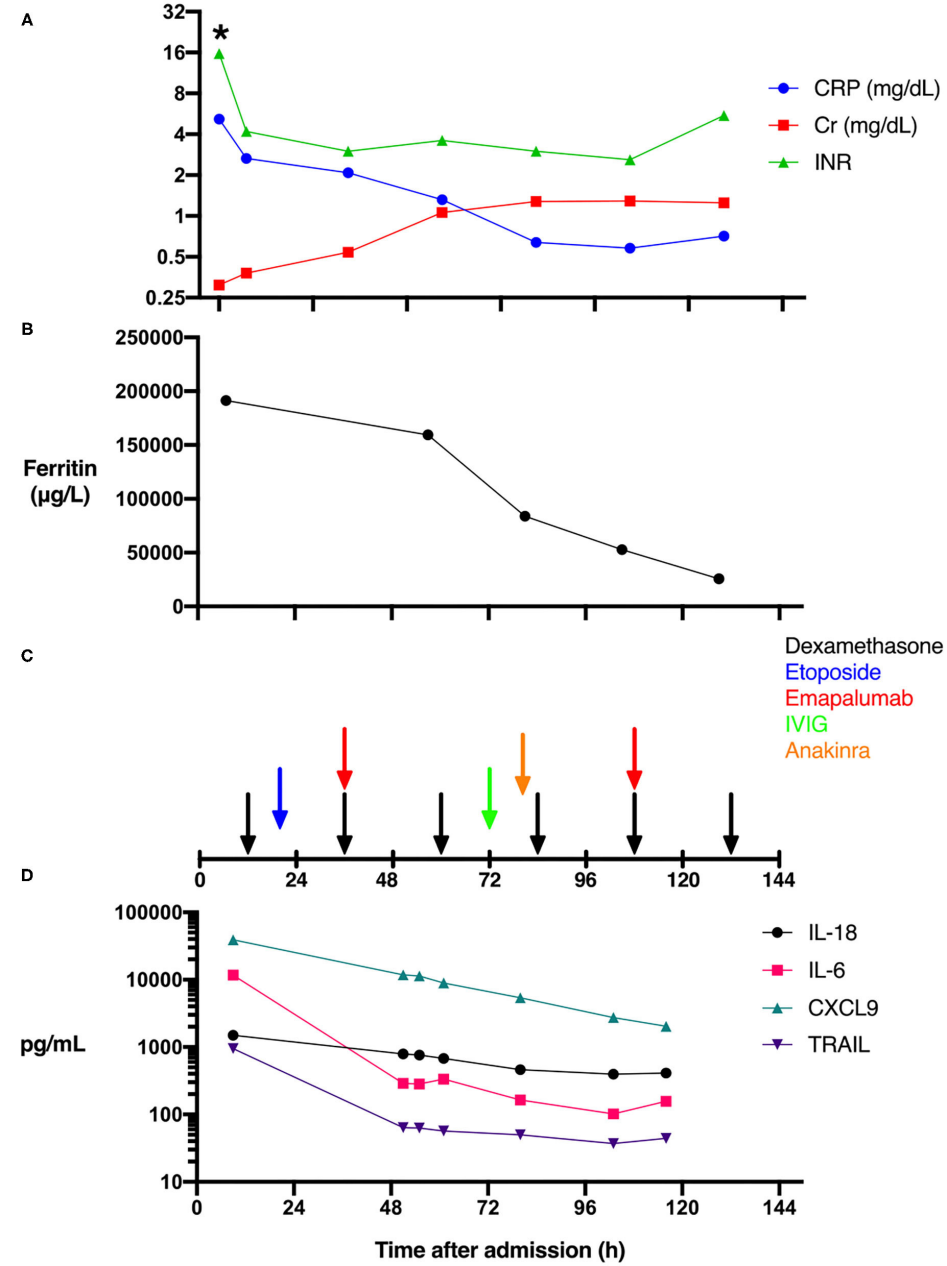
Effect of immune modulation on biomarkers, organ dysfunction, and cytokine levels during HSV-associated secondary HLH. The time course of clinical laboratory values of serum C-reactive protein (CRP), creatinine (Cr), and INR levels **(A)**, ferritin levels **(B)**, as well as the timing of the administration of the immune suppressive agents is shown **(C)**. While IL-6 and TRAIL levels fell precipitously after admission, CXCL9 and IL-18 levels fell more gradually **(D)** (*Actual INR resulted as >15.7).

The patient is an 11-day-old female infant born at 38 weeks gestational age to a G1P0 mother. The patient was referred to a local emergency department for increased work of breathing during her mother's routine postpartum evaluation. She was transported to our facility and quickly upgraded to the pediatric intensive care unit (PICU) due to lethargy, hypothermia, respiratory distress, and persistent bleeding. With a presumptive diagnosis of sepsis, laboratory workup including pan cultures was obtained and empiric antimicrobial therapy initiated with ceftazidime, ampicillin, and acyclovir at standard meningitic dosage. Initial laboratory findings were significant for transaminitis with an alanine aminotransferase (ALT) measured at 3,174 IU/L, and a total bilirubin level of 3.6 mg/dL. A complete blood count was only remarkable for thrombocytopenia (17,000 cells/μL). Further laboratory investigation demonstrated a severe coagulopathy with INR > 15.7 (Figure 1A) and fibrinogen <60 mg/dL. A mixed metabolic and respiratory acidosis was present based on venous pH 7.23, pCO2 56.4 mmHg, and elevated lactate 11.1 mmol/L. Inflammatory biomarkers revealed a slightly elevated C-reactive protein (CRP) of 5.18 mg/dL (Figure 1A) and procalcitonin of 0.65 ng/mL. Given the degree of fulminant hepatic failure, the medical team suspected hemophagocytic lymphohistiocytosis (HLH) and a ferritin level sent 6 h after admission measured 191,420 μg/L (Figure 1B). The remainder of the HLH diagnostic laboratory tests were also sent but bone marrow aspiration was not performed due to coagulopathy and general clinical

instability. Based on the extreme hyperferritinemia, the patient was presumptively diagnosed with HLH while the rest of the diagnostic workup continued.

Immune modulation with dexamethasone was begun 8 h after admission (Figure 1C). Given the medical team's previous experience with a very similar case (1), and the recent U.S. Food and Drug Administration (FDA) approval of emapalumab (human anti-IFN-γ antibody, SOBI, Sweden), for the treatment of primary HLH (2), the decision was made to administer emapalumab to block the inflammatory cascade. While waiting for emapalumab to arrive, etoposide was administered as per HLH-94 treatment protocol (3).

The emapalumab study protocol for primary HLH called for an initial dose of 1 mg/kg, followed by increased subsequent dosages of 3, 6, and up to 10 mg/kg. Extreme ferritin elevation has been significantly correlated with monokine induced by gamma interferon (MIG/CXCL9) levels (4). We therefore chose a moderate starting dose of emapalumab (6 mg/kg), which was administered 35 h after admission. Additional immune modulatory therapies were given including anakinra and intravenous immune globulin. Unfortunately, MODS progressed as evidenced by anuria and a rising serum creatinine (Figure 1A), as well as coagulopathy relatively refractory to the administration of fresh frozen plasma (Figure 1A), presumably due to loss of liver synthetic function and ongoing disseminated intravascular coagulation. The patient developed extreme fluid overload but was deemed ineligible for hemodialysis catheter placement due to small size (2.5 kg). On hospital day 6, after discussing the likely futility of ongoing treatment, parents requested that care be directed toward comfort measures only. She was compassionately extubated and expired in her mother's arms. Serum testing for herpes simplex virus 1 (HSV-1) by polymerase chain reaction returned positive shortly after the patient succumbed to her illness. The remainder of infectious workup was negative.

The patient initially fulfilled four of eight diagnostic criteria for HLH (3): fever (temperature instability), cytopenias (hemoglobin and platelets), hypofibrinogenemia, and hyperferritinemia. The soluble interleukin (IL)-2 receptor level measured 4,306 pg/mL (reference range 175.2–858.2 pg/mL), which met the five criteria threshold for diagnosing HLH. Circulating natural killer (NK) cell numbers were too low to reliably measure NK cell activity (5). Subsequent genetic analysis did not reveal any variants of clinical significance for the genes associated with familial HLH. Therefore, final diagnosis was secondary HLH due to acute primary disseminated HSV-1 infection. The final diagnosis and genetic testing results, as well as implications for future pregnancy planning, were discussed with the patient's family.

Additional cytokine and chemokine analysis was performed postmortem as the patient was enrolled in the authors' own Institutional Review Board (IRB)-approved observational study of sepsis immune phenotypes. Our aggressive immune modulatory therapies (Figure 1C) may have decreased IL-6 levels, as they fell precipitously from 11,713 to 289 pg/mL following the initiation of dexamethasone and etoposide (Figures 1C,D). In contrast, IL-18, which has been associated with macrophage activation syndrome (6), remained modestly elevated and relatively unaffected (Figures 1C,D). CXCL9, a serum biomarker of IFN-γ activity (7), was markedly elevated at 39,184 pg/mL, then gradually decreased with immunosuppressive therapy (Figures 1C,D). Lastly, we measured TNF-related apoptosis-inducing ligand (TRAIL) as a marker of immune response to viral infection. TRAIL levels were elevated at 948 pg/mL and demonstrated a robust decrease similar to IL-6 after suppressive therapy (Figures 1C,D).

## Discussion

In this case, we attempted to use the IFN-γ antagonist emapalumab to quell the pathogenic inflammatory cascade in a critically ill infant with secondary HLH due to disseminated HSV-1 infection. We initially believed this to be the first described use of emapalumab for secondary HLH. However, Dr. Jordan's group published the case of a 20-month-old boy with secondary HLH initially due to acute Epstein-Barr virus (EBV) infection, who subsequently developed multiple other viral and fungal infections after standard HLH-94 therapy (8). That patient was not enrolled in the existing emapalumab treatment trial due to multiorgan failure, but was instead treated using an emergency investigational new drug application as a last resort. While that patient survived, our patient ultimately succumbed to MODS and fulminant liver failure. Unfortunately, her small size limited our ability to provide dialysis as bridging support for the possibility of liver transplantation (9). We chose to administer etoposide as per the HLH-94 treatment protocol. Etoposide likely serves to deplete activated T and NK cells (10). However, etoposide treatment can be hepatotoxic (11) and neonatal dosing (12), especially in the setting of liver failure (13), is difficult. The use of more precise immunomodulatory therapies with fewer adverse effects on organ function would provide a safer treatment strategy for patients with MODS.

Disseminated HSV is commonly associated with immune deficiencies, as cytotoxic NK cells and T lymphocytes are crucial for the host immune response to HSV infection (14). This case highlights that in neonates, even with appropriate early antiviral treatment and immune suppression, disseminated HSV continues to have poor outcomes (15). Neonates have a quantitative immune deficiency due to low numbers of cytotoxic lymphocytes (16) and increased regulatory T cells (17). The relationship between HSV infection and HLH has been increasingly recognized (1, 18–20); however, controversy remains about whether this presentation truly represents HLH or is an “HLH mimic.” Upon presentation, the patient was already in fulminant hepatic failure, so the authors decided to treat with immune suppression in an effort to mitigate further organ damage. However, the exact pathophysiologic role of IFN-γ in this situation is still not clear. Some studies have demonstrated that IFN-γ is helpful in the initial clearance of HSV through synergy with type I IFNs (21, 22). However, other studies have shown that IFN-γ governs a limited role in the control of HSV and HSV pathogenesis (23, 24). Our use of emapalumab was based on the known role of IFN-γ in HLH.

IFN-γ was first implicated in the pathogenesis of HLH via the murine model of type 2 familial HLH: lymphocytic choriomeningitis virus (LCMV) infection of perforin-deficient (*prf1*^−/−^) mice (25, 26). This preclinical work led to the development and trial of emapalumab for primary HLH (2). The sources of IFN-γ secretion during HLH are antigen-specific CD8+ and CD4+ T cells as well as NK cells. This IFN-γ stimulates the activation of macrophages, and increases antigen presentation by both hematopoietic and non-hematopoietic cells. Fortunately, the adverse effect profile of emapalumab is minimal (2). Therefore, targeted blockade of IFN-γ with emapalumab is an attractive therapeutic strategy to block the inflammatory cascade in an attempt to avoid cytotoxic chemotherapy in the setting of MODS.

The decision to block IFN-γ may provide both benefits and drawbacks in a patient with a disseminated viral infection. If IFN-γ plays a role in suppressing HSV infection, blocking this pathway in the setting of an active viral infection could have deleterious effects. A large body of preclinical studies have examined the role of IFN-γ and IFN-γ receptor signaling in HSV infections in mice. Most of these studies examined HSV encephalitis and ocular infection, some examined vaginal (27) or mucosal infection (28), and a limited number investigated disseminated HSV infection (24). On one hand, several studies indicate that IFN-γ suppresses HSV (27), likely through synergy with type I IFNs acting to block HSV replication (21, 22) and reactivation (29). IFN-γ may also protect neurons from apoptosis (30). However, a large number of murine studies indicate that IFN-γ plays only a limited role in HSV systemic spread (24) and viral clearance (23, 28, 31–33). The role of IFN-γ during HSV is likely nuanced and depends on multiple factors including the mouse strain background and the virus subtype.

IFN-γ may not be the sole immune activator driving HLH pathogenesis. Another established model of HLH, murine cytomegalovirus (MCMV) infection of BALB/c mice, is not dependent upon IFN-γ (34). Additionally, sequential toll-like receptor (TLR) stimulation recapitulates HLH in mice by inducing a unique metabolic profile in macrophages but does not require IFN-γ. In another model of MAS/HLH (35), repeated TLR9 stimulation of C57BL/6 mice with CpG oligodeoxynucleotides induces HLH, though the full disease phenotype is only seen with blockade of the regulatory cytokine IL-10 (36). Interestingly, in the mouse cytomegalovirus (MCMV) model of herpes infection, IL-10 is chiefly produced by NK cells in the liver and provides protection from collateral injury by modulating the inflammatory response associated with MCMV infection (37, 38). One therapeutic avenue that was not explored in this case is the janus-associated kinase (JAK) inhibitor, ruxolitinib, which was shown to be superior to IFN-γ blockade in both primary and secondary mouse models of HLH due to its suppressive effects on activated T cells and neutrophils (39). Ruxolitinib, in addition to dexamethasone and etoposide, is being investigated for the treatment of newly diagnosed or refractory HLH.

Secondary HLH is likely a common final pathway of multiple disease processes. In this case, secondary HLH was the result of disseminated HSV infection. While emapalumab was not able to rescue this patient, perhaps earlier IFN-γ blockade, or other immune suppressive therapy, may have mitigated some of the organ damage without injurious side effects. Further investigation into whether early administration of targeted immune modulators for secondary HLH is warranted.

## Data Availability Statement

The original contributions presented in the study are included in the article/supplementary material, further inquiries can be directed to the corresponding author/s.

## Ethics Statement

The studies involving human participants were reviewed and approved by Penn State University Institutional Review Board. Written informed consent to participate in this study was provided by the participants' legal guardian/next of kin. Written informed consent was obtained from the minor(s)' legal guardian/next of kin for the publication of any potentially identifiable images or data included in this article.

## Author Contributions

DM and EH conceptualized the study, drafted the initial manuscript, and reviewed and revised the manuscript. TD and RK critically reviewed manuscript for important intellectual content, and reviewed and revised the manuscript. All authors approved the final manuscript as submitted and agree to be accountable for all aspects of the work.

## Conflict of Interest

The authors declare that the research was conducted in the absence of any commercial or financial relationships that could be construed as a potential conflict of interest.

## Abbreviations

HLH: Hemophagocytic lymphohistiocytosis
HSV: Herpes simplex virus
MODS: Multiple organs dysfunction syndrome.
